# Efficacy of integrative Traditional Chinese and Western medicine for the treatment of patients infected with 2019 novel coronavirus (COVID-19): A protocol for systematic review and meta analysis

**DOI:** 10.1097/MD.0000000000020781

**Published:** 2020-07-17

**Authors:** Dan Liu, Yanyan You, Yunhui Chen, Songqi Tang

**Affiliations:** a West China Hospital, Sichuan University, South Renmin Road, Wu Hou District, Chengdu; b Chengdu University of Traditional Chinese Medicine, Chengdu, China; c College of Traditional Chinese Medicine, Hainan Medical University, Haikou, China.

**Keywords:** Coronavirus disease 2019, integrative Traditional Chinese and Western medicine, meta-analysis, protocol, systematic review

## Abstract

**Background:**

No specific anti-virus drugs or vaccines have been available for the treatment of COVID-19. Integrative traditional Chinese and western medicine has been proposed as a therapeutic option with substantial applications in China. This protocol is proposed for a systematic review and meta-analysis that aims to evaluate the efficacy of integrative traditional Chinese and western medicine treatment on patients with COVID-19.

**Methods:**

Ten databases including PubMed, EMBASE, Cochrane Library, CIHAHL, Web of Science, Chinese National Knowledge Infrastructure (CNKI), Chinese Scientific Journals Database (VIP), Wanfang database, China Biomedical Literature Database (CBM) and Chinese Biomedical Literature Service System (SinoMed) will be searched. All published randomized controlled trials, clinical controlled trials, case-control, and case series that meet the pre-specified eligibility criteria will be included. Primary outcome measures include mortality, clinical recovery rate, duration of fever, progression rate from mild or moderate to severe, improvement of symptoms, biomarkers of laboratory examination and changes in computed tomography. Secondary outcomes include dosage of hormonotherapy, incidence and severity of adverse events and quality of life. Study selection, data extraction and assessment of bias risk will be conducted by 2 reviewers independently. RevMan software (V.5.3.5) will be used to perform data synthesis. Subgroup and sensitivity analysis will be performed when necessary. The strength of evidence will be assessed by the GRADE system.

**Results:**

This study will provide a well-reported and high-quality synthesis on the efficacy of integrative traditional Chinese and western medicine treatment on patients with COVID-19.

**Conclusion:**

This systematic review protocol will be helpful for providing evidence of whether integrative traditional Chinese and western medicine treatment is an effective therapeutic approach for patients with COVID-19.

**Ethics and dissemination:**

Ethical approval is unnecessary as no individual patient or privacy data is collected. The results of this study will be disseminated in a peer-reviewed scientific journal and/or conference presentation.

**Systematic review registration:**

PROSPERO CRD42020167205.

## 1 Introduction

Coronavirus disease 2019 (COVID-19) is an infectious disease caused by a novel severe acute respiratory syndrome coronavirus 2 (SARS-CoV-2).^[1–3]^ Most COVID-19 patients present with mild or uncomplicated illness, while approximately 14% of the infected progress to severe disease that requires hospitalization and oxygen support and 5% needs admission to an intensive care unit.^[4–6]^ Up to date, no efficient vaccines and specific anti-SARS-CoV-2 agents are available to prevent or treat the disease, symptomatic and supportive treatments are main strategies to manage the infection.^[7–9]^

Traditional Chinese medicine (TCM) integrated with western (conventional) medicine has been proposed as a therapeutic option for COVID-19 in China.^[10–12]^ By 23 March 2020, a total of 74,187 confirmed cases in China (91.5% of total confirmed cases) have been treated with TCM and the total effective rate reached over 90%.^[13]^ The available data indicate that integrating TCM with western medicine can alleviate clinical symptoms, decrease duration of fever, facilitate radiological improvement, prevent mild cases from developing into severe cases, shorten the length of hospital stay and reduce fatality rate.^[14–16]^

A systematic review and meta-analysis can provide clear evidence regarding the benefits of certain healthcare intervention. Herein, we propose a protocol for a systematic review and meta-analysis to evaluate the efficacy of integrative traditional Chinese and western medicine on COVID-19. The findings of this study may yield helpful evidence for the patients, clinicians, investigators and policymakers concerned about the efficacy of integrative traditional Chinese and western medicine on COVID-19.

## 2 Methods

### 2.1 Study registration

This systematic review protocol has been registered on PROSPTERO (www.crd.york.ac.uk/prospero/) with number PROSPERO CRD42020167205. Ethical approval is unnecessary because this study only involves the data of previous studies.

### 2.2 Eligibility criteria

#### 2.2.1 Type of study

Randomized controlled trials (RCTs), clinical controlled trials (CCTs), case-control and case series of integrative traditional Chinese and western medicine treatment for COVID-19 will be included. Animal-based research and literature review will be excluded.

#### 2.2.2 Participants

Patients diagnosed with COVID-19 using any recognized diagnostic criteria will be included regardless of the age, gender and source of cases and the duration and severity of disease. Patients infected with adenovirus, rhinovirus, human metapneumovirus, etc., will be excluded.

#### 2.2.3 Types of interventions

The intervention group will be treated by integrative traditional Chinese and western medicine. TCM in the study is defined as herbal formula and patent medicine. There will be no restriction regarding conventional western medical regimen (such as supportive treatment, IFN-α, lopinavir or ritonavir).

#### 2.2.4 Types of comparator(s)/control

The control group will be treated with the same conventional western medical regimen as the intervention group in the same original study. No restrictions are imposed regarding conventional western medicine treatment regimen. Studies comparing different traditional Chinese medicine will be excluded.

#### 2.2.5 Types of outcome measures

Primary outcome measures include mortality, clinical recovery rate, duration of fever, progression rate from mild or moderate to severe, improvement of symptoms, biomarkers of laboratory examination and changes in computed tomography. Secondary outcomes include dosage of hormonotherapy, incidence and severity of adverse events and quality of life.

### 2.3 Information source

Ten databases including PubMed, EMBASE, Cochrane Library, CIHAHL, Web of Science, Chinese National Knowledge Infrastructure (CNKI), Chinese Scientific Journals Database (VIP), Wanfang database, China Biomedical Literature Database (CBM) and Chinese Biomedical Literature Service System (SinoMed) will be searched from the inception to 1 March 2021. Studies from Clinical Trials.gov (http://www.clinical.trails.gov), Chinese Clinical Trial Registry (http://www.chictr.org/cn/) and WHO international Clinical Trial Registry Platform (https://www.who.int/ictrp/en/) will also be searched. The reference lists of the retrieved articles will be manually reviewed for further additional trials. Corresponding author will be contacted for incomplete data.

### 2.4 Search strategy

Two reviewers will search the literature independently. Any inconsistency will be solved by a third reviewer. Manual search will be performed for relevant studies found in the reference lists of included studies. The electronic search will be conducted using a combination of following terms: novel coronavirus, Severe Acute Respiratory Syndrome Coronavirus 2, SARS-CoV-2, COVID-19, COVID19, 2019-nCoV, integrative traditional Chinese and western medicine, integrated traditional Chinese and western medicine, integrative Chinese and western medicine, integrated Chinese and western medicine, Traditional Chinese medicine, Chinese medicine, Chinese traditional medicine, complementary and alternative medicine, phytotherapy, herbal medicine, oriental medicine, randomized controlled trial, controlled clinical trial, randomized, randomly, trials, case-control, case series, CCT, and RCT. The search strategy for PubMed is presented in Table 1 and will be modified in other databases.

**Table 1:**
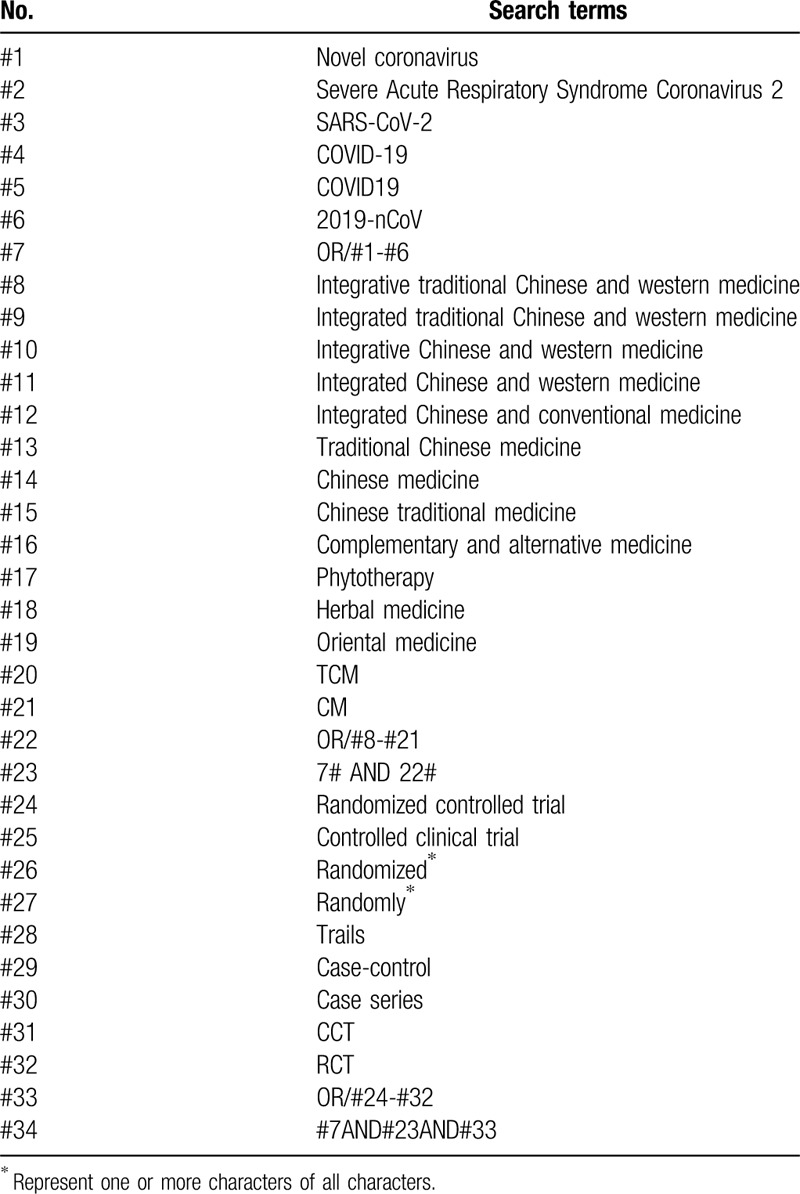
Search strategy for the PubMed.

### 2.5 Data collection and analysis

#### 2.5.1 Study selection

**Figure 1:**
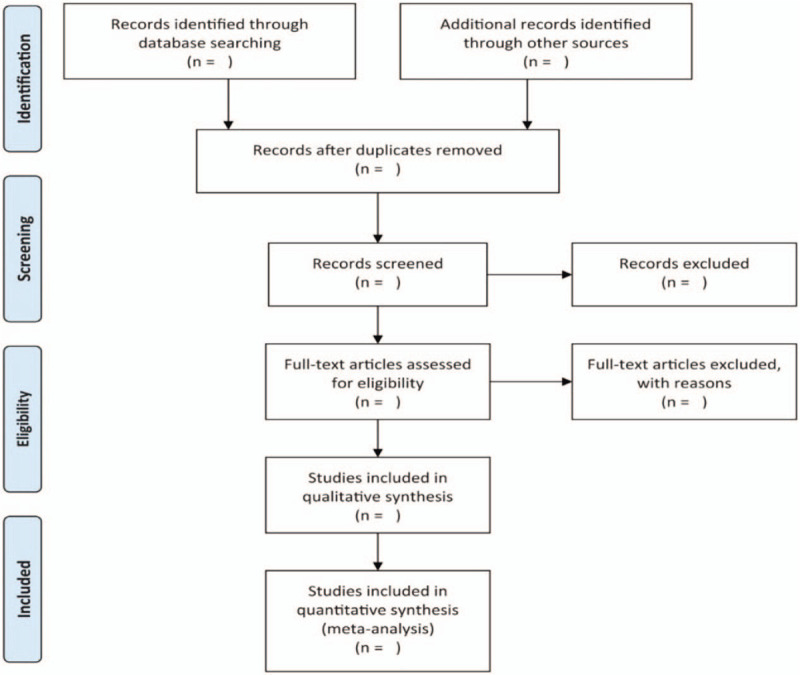
Flowchart of study selection.

Two reviewers will perform screening, study selection and data extraction independently. The literature obtained will be imported into EndnoteX9 to screen the title and abstract, the duplications and studies failing to meet the pre-specified inclusion criteria will be excluded. After reading the full text of the remaining studies, the final included studies will be determined. The corresponding author from any original study will be contacted when the full text is unavailable. Any disagreements will be arbitrated by a third reviewer. The entire process of study selection is presented in a PRISMA flow chart (Fig. 1).

#### 2.5.2 Data extraction and management

The following data will be extracted by 2 reviewers independently from eligible studies and input into a pre-specified data acquisition form: reference ID, author information, year of publication, study type, study design, setting of study, sample size, participant characteristics (age, gender, duration and severity of illness, laboratory test, CT scan, etc.), integrative intervention group and control group (details of randomization, blinding, allocation, intervention approach and duration), and primary and secondary outcomes at all reported time points. Inconsistency between two reviewers will be solved by a third reviewer. All data will be cross-checked and transferred to RevMan software (V.5.3).

#### 2.5.3 Assessment of risk of bias

Two reviewers will assess the risk of publication bias for every included study using the Cochrane risk of bias assessment tool independently in terms of eight domains, namely randomization sequence generation, randomization allocation concealment, blinding of participants, blinding of personnel, blinding of outcome assessors, incomplete outcome data, selective reporting bias and other bias. Each domain will be graded as high, unclear, or low risk of bias.^[17]^ Corresponding authors will be contacted for unclear domains. Inconsistency will be solved by consultation with a third reviewer.

#### 2.5.4 Measures of treatment effect

Efficacy data will be synthesized and statistically analyzed by 2 reviewers independently using RevMan 5.3. A risk ratio or odd ration with 95% CIs will be adopted for dichotomous data, whereas a mean difference (MD) or standard mean difference (SMD) with 95% CIs will be utilized for continuous data. SMD will be employed if different assessment tools are used.

#### 2.5.5 Dealing with missing data

If required data is unclear or missing, reviewers will contact the corresponding author of the original study by E-mail or telephone. If data is still unattainable, the study concerned will be excluded from the analysis. A sensitivity analysis will be performed to address the potential impact of missing data.

#### 2.5.6 Assessment of heterogeneity

Statistical heterogeneity will be investigated using χ^2^ test and *I*^2^ statistic. Fixed-effect model will be applied when heterogeneity is low (*I*^2^ < 50%) and random-effects model will be used for moderate heterogeneity (50% < *I*^2^ < 75%). When heterogeneity is considerably high, meta-analysis will not be performed.

#### 2.5.7 Assessment of reporting biases

Funnel plots will be performed to assess potential reporting bias when more than 10 studies are included. In additional, Egger regression and Begg correlation test will be conducted to identify the funnel plot asymmetry.

#### 2.5.8 Data synthesis

In line with the Cochrane guideline, the fixed-effects model will be utilized for the pooled data if heterogeneity is deemed low and the random-effect model will be employed if heterogeneity is deemed moderate. Subgroup analysis or meta-regression will be performed to assess the potential sources with reasonable explanations if heterogeneity is considerably high. The statistical significance is defined as *P* < .05. If the meta-analysis is not feasible, a narrative description of the results will be provided.

#### 2.5.9 Subgroup analysis and investigation of heterogeneity

If feasible, subgroup analyses will be performed in terms of severity of included patients, duration of disease, routes of administration, dosage, preparations and ingredients of TCM interventions. Subgroup analyses will be conducted to interpret the heterogeneity.

#### 2.5.10 Sensitivity analysis

If feasible, sensitivity analysis will be conducted to evaluate the robustness of the pooled effects of the included studies given impact of such variables as sample size, methodological quality, missing data or high risk of bias.

#### 2.5.11 Grading the quality of evidence

The Cochrane Collaboration Network GRADE (The Grading of Recommendations Assessment Development and Evaluation) will be utilized to grade the quality of evidence as very low, low, moderate or high.^[18,19]^ The quality of evidence of a specific study will be assessed according to the risk of bias, imprecision, inconsistency, indirectness, publication bias, effect size or dose-response relation. The findings will be presented with A Summary of Finding table. Any discrepancy will be arbitrated by discussion or a third reviewer.

## 3 Discussion

Chinese patent medicine and herbal formulas such as *Jinhua Qinggan* Granule, *Lianhua Qingwen* Capsule, *Xuebijing* Injection, *Qingfei Paidu Tang*, *Huashi Baidu Fang*, *Xuanfei Baidu Fang*, *Maxing Ganshi Tang*, *Yinqiao San*, *Mahuang Shengma Tang*, *Xiao Chaihu Tang*, *Da Yuan Yin*, *Gancao Ganjiang Tang* and *Shegan Mahuang Tang* have been prescribed based on pattern differentiation. Existing clinical evidence indicate that integrative traditional Chinese and western medicine might be a potent therapeutic approach for COVID-19. Herein, we propose a protocol for a systematic review and meta-analysis to evaluate the efficacy of integrative traditional Chinese and western medicine on COVID-19.

All RCTs, CCTs, case-control and case series regarding the integrative traditional Chinese and western medicine will be fully considered and synthesized without language or publication restrictions. This meta-analysis will provide a relatively convincing conclusion of whether integrating TCM with western medicine is effective for treating patients with COVID-19. Conclusions drawn from this review may benefit patients, clinicians, investigators and policymakers. The process of conducting this review will be divided into identification, study inclusion, data extraction and data synthesis. If amendments to this protocol are necessary, the date of each amendment with statement of the changes and the corresponding reasons will be provided.

## Acknowledgments

A special acknowledgement goes to all researchers who registered for clinical trials and to all the medical staff fighting against COVID-19.

## Author contributions

**Conceptualization:** Songqi Tang, Yunhui Chen.

**Formal analysis:** Dan Liu, Yanyan You, Yunhui Chen.

**Investigation:** Dan Liu, Yanyan You, Yunhui Chen.

**Methodology:** Dan Liu, Songqi Tang, Yunhui Chen.

**Validation:** Songqi Tang, Yunhui Chen.

**Visualization:** Dan Liu.

**Funding acquisition:** Songqi Tang.

**Writing – original draft:** Dan Liu, Yanyan You.

**Writing – review & editing:** Songqi Tang, Yunhui Chen.
